# Impact of Psychiatric Rehabilitation on Chronicity and Health Outcomes in Mental Disorders: A Quasi-Experimental Study

**DOI:** 10.3390/healthcare14020250

**Published:** 2026-01-20

**Authors:** Marta Llorente-Alonso, Marta Tello Villamayor, Estela Marco Sainz, Pilar Barrio Íñigo, Lourdes Serrano Matamoros, Irais Esther García Villalobos, Irene Cuesta Matía, Andrea Martínez Abella, María José Velasco Gamarra, María Nélida Castillo Antón, María Concepción Sanz García

**Affiliations:** 1 Faculty of Health Science, University of Valladolid, 42005 Soria, Spain; 2 Gerencia de Asistencia Sanitaria del Área de Salud de Soria, Complejo Hospitalario de Soria, Gerencia Regional de Salud de Castilla y León (Sacyl), Pº Santa Bárbara s/n, 42005 Soria, Spain; mtellov@saludcastillayleon.es (M.T.V.); emarco@saludcastillayleon.es (E.M.S.); mpbarrioi@saludcastillayleon.es (P.B.Í.); lserrano@saludcastillayleon.es (L.S.M.); iegarciav@saludcastillayleon.es (I.E.G.V.); icuestama@saludcastillayleon.es (I.C.M.); amartinezab@saludcastillayleon.es (A.M.A.); mjvelascog@saludcastillayleon.es (M.J.V.G.); ncastillo@saludcastillayleon.es (M.N.C.A.); msanzgar@saludcastillayleon.es (M.C.S.G.)

**Keywords:** psychiatric rehabilitation, mental illness, chronic disease, quality of life, health outcomes, hospitalization, partial

## Abstract

**Background/Objectives**: People suffering from mental illnesses are more likely to experience adverse social and health outcomes. Various interventions have been shown to help people with mental illness achieve better results in terms of symptom reduction, functional status, and quality of life. Psychiatric rehabilitation interventions integrate evidence-based practices, promising approaches, and emerging methods that can be effectively implemented to enhance health outcomes in this population. This study aims to examine whether the rehabilitative treatment provided to a group of patients with mental illness leads to improvements in health outcomes and psychiatric symptomatology. **Methods**: This study employed a retrospective quasi-experimental design. Data were collected between 2023 and 2025 within the Partial Hospitalization Program of the Psychiatry and Mental Health Service of Soria (Spain). The sample consisted of 58 participants who received rehabilitative treatment in this setting. Data were collected at the time of patients’ admission and at discharge. Gender, age, psychiatric diagnosis according to ICD-10, and the average length of stay in the rehabilitation program were assessed. The questionnaires administered were psychometrically validated scales related to heteroaggressiveness, perceived quality of life, global functioning, attitudes toward medication, and the risk of suicide. **Results**: A significant improvement was observed in the Global Assessment of Functioning (GAF) Scale (*t* = −7.1, *p* < 0.001), with mean scores increasing from 42.17 at admission to 69.13 at discharge. Additionally, reductions in suicidal risk and hetero-aggressive behavior were noted, alongside improvements in quality of life and treatment adherence. **Conclusions**: The findings highlight the effectiveness of implementing activities and programs focused on psychiatric rehabilitation processes to promote positive health outcomes. Future research directions and practical implications are discussed to support the continued development and optimization of psychiatric rehabilitation programs.

## 1. Introduction

Mental disorders constitute a major global public health concern. According to the World Health Organization, “in 2019, one in eight individuals worldwide, approximately 970 million people, were living with a mental disorder” [1]. Furthermore, the Spanish Mental Health Confederation in 2023 projects that mental health problems will become the leading cause of disability worldwide by 2030 [2]. According to the 2023 Annual Report of the Spanish National Health System, 34% of the Spanish population experiences some form of mental health problem. Mental health issues exceed 40% in people over 50 years of age and 50% in those over 85. The most prevalent mental disorders are anxiety disorders, followed by sleep disorders and depressive disorders [3]. According to the Spanish Ministry of Health, schizophrenia has an overall prevalence of 0.37% in the general population. It is more frequent in men (0.45%) than in women (0.29%). The sex-related prevalence difference is most pronounced between ages 20 and 49, while rates equalize after age 65. Additionally, a marked social gradient is observed, with higher prevalence in lower-income groups [4].

Although mental disorders encompass a wide range of conditions, it is important to emphasize the availability of effective strategies for both prevention and treatment [1]. Individuals living with mental illness are at increased risk of experiencing adverse social and health outcomes. Multiple meta-analyses have demonstrated that specific interventions can lead to meaningful improvements in symptom severityamong people with mental disorders [5]. In this context, specialized psychiatric rehabilitation services play a pivotal role in enhancing recovery trajectories.

Psychiatric rehabilitation units are specialized components of mental healthcare systems, designed to support recovery and promote the social, occupational, and community reintegration of individuals with severe and persistent mental disorders. As noted by the World Health Organization (WHO) in 2021, these units deliver structured programs that integrate clinical treatment with psychosocial and occupational support, social skills training, and interventions intended to foster autonomy, treatment adherence, and quality of life [6].

In Spain, public services focused on psychiatric rehabilitation comprise a heterogeneous network of care models that differ in their level of clinical intensity, length of stay, degree of community integration, and target population. These include Partial Hospitalization and Day Hospital programs, Psychiatric Rehabilitation Units, Psychiatric Convalescence Units, Psychosocial Rehabilitation Centers, and Rehabilitation Units for individuals with severe and persistent mental illness [7].

Their overarching aim is to enable individuals with mental illness to manage symptoms, overcome interpersonal and environmental barriers associated with disability, regain independent living skills, engage socially, manage daily life effectively, and recognize personal limitations [8]. These units incorporate a combination of evidence-based interventions, promising practices, and emerging therapeutic approaches that help individuals with severe mental illness choose, obtain, and maintain valued social roles, complemented by targeted psychosocial treatment modalities. Collectively, these components constitute a comprehensive framework that facilitates recovery [9].

In contrast to acute inpatient psychiatric units, primarily oriented toward symptomatic stabilization, psychiatric rehabilitation units focus on functional recovery and the acquisition of personal, social, and vocational competencies. According to the American Psychiatric Association in2022, these interventions commonly include patient and family psychoeducation, cognitive rehabilitation, occupational therapy, and community-based recovery initiatives that promote social inclusion and reduce stigma [10].

In the province of Soria (Spain), a Partial Hospitalization Program (PHP) is available, with a maximum capacity of 20 patients attending from Monday to Friday during morning hours. The program is specifically designed for patients referred from Mental Health Teams or Hospitalization Units, whose clinical needs necessitate medium-term care. The objectives of the program include providing an alternative to full hospitalization, thereby minimizing, to the extent possible, the separation of the patient from their family and social environment. Additionally, the program aims to facilitate discharges from Crisis Hospitalization Units and offers support, supervision, and monitoring during the transition from hospital to home, ensuring a gradual reintegration into community life. Furthermore, it delivers treatments requiring a higher level of care that cannot be provided by Mental Health Teams [7].

The present study aims to analyze whether the rehabilitative treatment provided to a cohort of individuals with mental illness is associated with significant improvements in health outcomes and psychiatric symptomatology.

The intervention implemented in the Partial Hospitalization Program (PHP) followed a comprehensive, primarily group-based psychotherapeutic model designed for patients experiencing psychopathological decompensation. A multidisciplinary team conducted an initial assessment of each patient and subsequently developed an individualized treatment plan focused on psychosocial rehabilitation. The program addressed key domains essential for adaptive functioning, including self-care, personal competence, health management, independence, and autonomy. Cognitive rehabilitation was a central component, aimed at restoring cognitive abilities such as attention, memory, concentration, perception, and spatiotemporal orientation through structured exercises and repetition-based training.

Interpersonal and behavioral functioning were also targeted. Patients acquired or recovered necessary behavioral repertoires for effective social interaction, and specific social skills training sessions were implemented. Psychoeducation was provided regularly, covering topics such as symptom recognition, the therapeutic benefits and side effects of medication, strategies to enhance treatment adherence, and the identification of appropriate healthcare resources for crisis situations. In addition, psychomotor functioning and physical well-being were supported through programs involving physical exercise, sports activities, and maintenance gymnastics. Social and community participation was reinforced by promoting the use of community resources, the development of social networks, and pathways toward socio-occupational reintegration. Family involvement was actively encouraged through guidance and support sessions. Finally, both individual and group leisure activities were facilitated to foster the development of hobbies and promote meaningful and enjoyable use of free time. The group sessions mentioned are shown in Table 1. Supplementary Materials include a document that explains in detail the activities of the partial hospitalization program.

On the other hand, research studies focused on psychiatric rehabilitation are heterogeneous regarding the variables examined and the objectives pursued. These differences are mainly due to the broad concept of mental health rehabilitation in research, but variations are also observed between studies depending on the country of origin and the healthcare systems operating in those countries [11].

Moreover, the studies analyzed in the present research highlight the need to provide more data regarding the health outcomes associated with these types of psychiatric units. For example, Zhang et al. evaluated two different types of rehabilitation (homestyle rehabilitation vs. hospital rehabilitation) and demonstrated that therapy improved global functioning scores in both groups. However, the homestyle group achieved higher scores in changes in social functioning compared to the hospital rehabilitation group, based on the assessment using the Global Assessment of Functioning (GAF) scale [12]. Regarding treatment adherence and perceived quality of life, it has been shown that the health education provided by psychiatric units is just as effective as a specific adherence therapy intervention [13].

Therefore, the overall objective of this study is to examine the improvements and changes in functioning, as well as in psychiatric symptomatology, among a group of individuals with mental illness undergoing rehabilitative treatment in a partial hospitalization program. Specifically, the aim is to analyze whether a rehabilitative intervention during treatment carried out in a partial hospitalization program reduces heteroaggressiveness, improves perceived quality of life, global functioning, attitudes toward medication, and decreases the risk of suicide.

## 2. Materials and Methods

### 2.1. Study Design

We completed a retrospective evaluation utilizing a pretest/posttest design to assess changes in recovery and health outcomes among people living with severe and persistent mental illnesses after receiving psychiatric rehabilitation services at the Partial Hospitalization Program of the Psychiatry and Mental Health Service of Soria (Spain). To evaluate the effectiveness of the programs, we collected and compared data from individuals with severe mental illness at admission to and discharge from the partial hospitalization program. The medical records of 58 patients admitted between October 2023 and October 2025 were reviewed. All participants were over 18 years of age and had a diagnosis of a severe and chronic mental illness, with a higher prevalence of personality disorders, schizophrenia spectrum disorders, and affective disorders. A non-probabilistic convenience sampling method was employed, including all patients admitted to this psychiatric rehabilitation facility during the study period. The study protocol was approved by the Research Ethics Committee of the Burgos and Soria Health Area (Approval No. 3092/2025).

### 2.2. Measures

Regarding demographic data, Psychiatric Diagnosis according to ICD-10, age, months of stay in the rehabilitation program and gender were recorded.

The instruments used in the present study were as follows:

- Buss–Durkee Hostility Inventory (BDHI): This scale consists of 75 True/False items distributed across eight subscales: Assault (Physical Aggression), Indirect Hostility, Irritability, Negativism, Resentment, Suspicion, Verbal Hostility, and Guilt [14]. Bishop and Quah reported an excellent Cronbach’s alpha for the total scale of 0.85 [15].
- Plutchik Suicide Risk Scale (PSRS): Developed by Robert Plutchik in 1989, this scale comprises 15 dichotomous (Yes/No) items. A total score of ≥ 6 (out of 15) was used as the cut-off point indicating suicide risk [16]. The Spanish validated version by Rubio et al. in 1998 was used in this study [17]. The original version reported a Cronbach’s alpha coefficient of approximately 0.84, while the Spanish validation reported an internal consistency (Cronbach’s alpha) of around 0.90.
- Global Assessment of Functioning (GAF): This scale is used to assess the overall level of psychological, social, and occupational functioning during the past month [18]. It is scored on a 0–100 scale, divided into 10-point intervals, where higher scores indicate better functioning and fewer psychiatric symptoms. It has shown good reliability and validity indicators in previous studies [19].
- World Health Organization Quality of Life—BREF (WHOQOL-BREF): The abbreviated version of the WHOQOL scale, composed of 26 items rated on a 5-point Likert scale, was used [20,21]. For the assessment of quality of life, the Psychological Health subdimension was selected, which is composed of 8 items.
- Drug Attitude Inventory-10 (DAI-10): This inventory includes 10 True/False items with scores ranging from −10 to +10, where higher scores indicate a more positive attitude toward medication. This measure directly influences the patient’s therapeutic adherence [22]. In the original version, the internal consistency (Cronbach’s alpha) was 0.81, indicating good reliability.

### 2.3. Data Analysis

The data obtained through the responses of the participants were processed and analyzed with the IBM SPSS Statistics v.30.0 (IBM Corp., Armonk, NY, USA), whose license was granted by the University of Valladolid [23].

The characteristics of the participants were analyzed via counts *(N)* and proportions (%) for qualitative variables and measures of central tendency (means (*M*) and standard deviation *(SD*) for quantitative variables. Descriptive and inferential analyses of the quantitative variables of the study will be carried out, reflecting the total number of subjects being compared at the two time points and their scores on the different questionnaires. The t-test was used to compare means between groups, with significance set at 0.05, to assess whether there were significant differences (*p* < 0.05) between the different groups. For all continuous outcomes, pre- and post-intervention differences were analyzed using paired-sample tests, applying either the paired Student’s t-test or the Wilcoxon signed-rank (W) test, as appropriate, and including only participants with complete data at both time points, in accordance with the within-subject quasi-experimental design. The normality test used for the continuous quantitative variables was the Shapiro–Wilk test and the Kolmogorov–Smirnov test, depending on the sample size.

Given the exploratory nature of this study and the limited sample size, no adjustment for multiple comparisons was applied. Therefore, *p*-values should be interpreted as exploratory rather than confirmatory.

## 3. Results

The participants were 58 individuals who received rehabilitative treatment between 2023 and 2025 in the partial hospitalization program of a hospital in Soria, Spain. Regarding gender, 32 were women (55.2%), 25 were men (43.1%), and 1 was a transgender person (1.7%). The mean age was 43.66 years (*SD* = 14.28). The youngest participant was 22 years old, and the oldest was 70, reflecting the heterogeneity of the sample. The average length of stay in the partial hospitalization program receiving rehabilitative treatment, measured in months, was 4.83 (*SD* = 5.84). The intervention followed a standardized, protocolized weekly structure. Each therapeutic module consisted of 20 scheduled sessions, typically delivered on a weekly basis. Post-intervention assessments were conducted at the completion of each module. Continuation to subsequent modules or discharge was determined based on clinical improvement relative to the therapeutic goals defined in the initial treatment plan. Although session-level attendance was not systematically recorded, all modules were delivered according to the predefined protocol.

**Figure 1:**
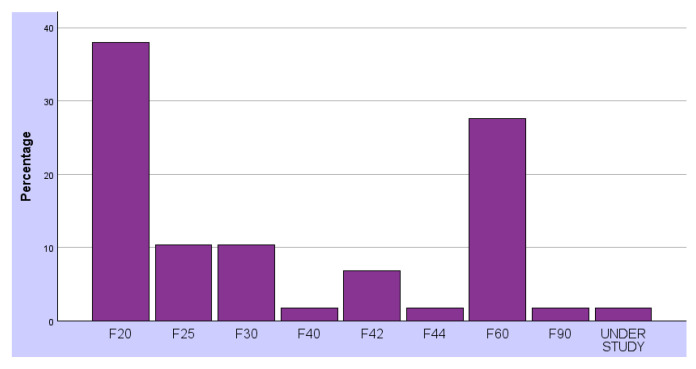
Percentage of Psychiatric Diagnosis according to ICD-10.

The psychiatric diagnoses reported among the 58 participants, according to the ICD-10, are as follows (see Figure 1 and Table 2):

− F20: Schizophrenia;
− F25: Schizoaffective disorder;
− F30: Mood (affective) disorders;
− F40: Anxiety and fear-related disorders;
− F42: Obsessive-compulsive and related disorders;
− F44: Dissociative disorders;
− F60: Personality disorder;
− F90: Hyperkinetic disorders.

All previously mentioned participants received psychopharmacological treatment and follow-up by their psychiatrist. The specific medications prescribed to each participant were not coded in this study. The main reason is that, during the prolonged stay in the rehabilitation program, adjustments in psychiatric medication were required for the patients. Nevertheless, the drugs administered to the patients according to their pathology included antidepressants, antipsychotics, anxiolytics and hypnotics, and mood stabilizers.

The descriptive analyses performed for the quantitative variables of the study reflect a significant loss of subjects, thus showing a lower number of participants in the second data collection (see Table 3).

First, normality tests were conducted on the paired sample, considering only those participants who were assessed at both pre and post-intervention time points. All variables showed a normal distribution except for the Global Assessment of Functioning (GAF) scale. The results are presented in Table 4.

We subsequently conducted inferential analyses using parametric tests (Student’s *t*-test) when the assumption of normality was met, and non-parametric tests (Wilcoxon signed-rank test) for variables that did not follow a normal distribution. Inferential tests were conducted on the paired subsets shown in Table 5. The number is much smaller than the questionnaires collected at baseline, and the reasons are as follows:

○ 20 participants were unable to complete the post-questionnaire, as the administration of the scales takes place at discharge and these patients are still admitted to the Partial Hospitalization Program.
○ 19 participants (32% of the sample) correspond to missing data. Of these, 10 declined to participate in the second data collection, citing fatigue from the first assessment. 9 correspond to patients who voluntarily left the unit before completing their rehabilitative therapy, most of whom had little or no awareness of their illness.

As shown in Table 5, statistically significant differences were found between the pre-rehabilitation assessment and the discharge assessment in the variables heteroaggressiveness (*t* = 2.12, *p* < 0.04), quality of life (*t* = −2.48, *p* < 0.01), and attitudes toward medication *(t* = −2.97, *p* < 0.004). Regarding suicide risk, although differences were observed between the two time points, these did not reach statistical significance.

With respect to the GAF, the *p*-value was lower than 0.05; therefore, the null hypothesis was rejected, indicating sufficient evidence to conclude that there are statistically significant differences in patients’ functional levels between 2023 and 2025, with a significance level of 5% (*Z* = −4.80, *p* < 0.001). The Global Assessment of Functioning scale had a larger sample size than the other questionnaires, with a total of 46 observations, as it is clinician-administered rather than self-administered, unlike the other scales included in the present study.

Cohen’s *d* and Hedges’ *g* are used to measure the effect size in comparisons between two groups before and after an intervention. Hedges’ g includes a correction for small-sample bias, which is why we included both measures in Table 6. On the other hand, we can observe that the effect sizes can be considered moderate.

For a comparison of means with related samples, analyzed using the Wilcoxon signed-rank test, the most commonly used effect size is r. The Wilcoxon signed-rank test showed a large effect size (*r* = −0.70), indicating a substantial change in Global Assessment of Functioning (GAF) scores between the pre and postintervention assessments. The negative sign reflects the direction of the observed change across time. Although this finding suggests a clinically meaningful improvement in overall functioning, the contribution of other concurrent factors cannot be ruled out. Hereafter, the formula used for its calculation is presented:

*r = Z/√n* = −4.80/√46 = −0.70

## 4. Discussion

The main objective of the present study was to analyze improvements and changes in functioning, as well as psychiatric symptomatology, in a group of individuals with mental illness undergoing treatment in a psychiatric rehabilitation unit. The findings indicate that, following a group-based rehabilitation intervention, global functioning, heteroaggressive behavior, quality of life, and attitudes toward medication improved significantly.

Regarding improvements in quality of life and global functioning, these results are consistent with previous research. Sachdeva and Sharma emphasized the relevance of rehabilitative interventions for enhancing quality of life and psychosocial adaptation among patients attending a psychiatric rehabilitation center [24]. Similarly, Kallivayalil and Sudhakar implemented a psychosocial rehabilitation program focused on occupational engagement, daily living skills, and group activities (e.g., cooking units, laundry, tailoring, crafts). After assessing patients’ subjective quality of life, they reported improvements across all domains (physical, psychological, environmental, and social relationships) [25]. Other authors have also documented that rehabilitation programs effectively induce positive changes among participants, such as increased perceptions of receiving help and enhanced usefulness of activities for achieving community-based employment [26].

On the other hand, it is important to emphasize the potential influence of other variables on the improvement of overall functioning, such as the use of psychotropic medications. Psychiatric medication acts as an adjunct to rehabilitative therapy, reducing symptoms that may hinder progress and improvements in health outcomes. Therefore, while the role of pharmacological treatment should not be overlooked, it is equally important to highlight the relevance of the activities carried out within the rehabilitation program in terms of habit formation, enhancement of social relationships, and promotion of self-care.

In relation to heteroaggressiveness, the present findings align with prior studies highlighting the essential role of rehabilitation programs in reducing aggressive and maladaptive behaviors. These effects are achieved through the development of emotional regulation strategies, interpersonal skills, and cognitive techniques aimed at conflict management [27].

Furthermore, the improvement observed in attitudes toward medication underscores the impact of psychoeducation and professional guidance on patients’ understanding of their illness and pharmacological treatment. Our results suggest that interventions promoting knowledge about symptoms, management of side effects, and the importance of treatment adherence help reduce negative perceptions associated with psychotropic medications. Sendt et al. reported a strong link between education, treatment understanding, and positive medication attitudes [28].

It is also important to note that, although patients reported a lower suicide risk, the improvement was modest and did not reach statistical significance. The literature on psychosocial interventions and rehabilitation programs presents mixed findings. While some studies report significant reductions in suicidal ideation or attempts [29], others observe only small, non-significant decreases [30]. The most robust effects are typically observed when interventions specifically target suicidal behavior (e.g., Dialectical Behavior Therapy) [31]. As in the present study, many investigations conducted in psychiatric rehabilitation settings report clinically meaningful changes that do not always achieve statistical significance. Thus, improvements may depend on the type of intervention, patient characteristics, follow-up duration, and sample size.

In summary, these findings highlight the effectiveness of implementing activities and programs centered on psychiatric rehabilitation processes to promote positive health outcomes.

### 4.1. Limitations

This study aimed to evaluate health outcomes following a psychiatric rehabilitation intervention in a Partial Hospitalization Program with a maximum capacity of 20 patients. Consequently, the most notable limitation is the small sample size, which may have reduced statistical power to detect effects of smaller magnitude. It is important to emphasize that the average length of stay in the partial hospitalization program is nearly 5 months, with a high standard deviation, which makes it challenging to collect data for research purposes. Furthermore, at this time, 20 individuals remain receiving rehabilitative treatment, which has prevented the administration of discharge questionnaires. Other studies found in the literature have shown the same limitation due to the small sample size [32]. It is important to note that, despite the inferential analyses presented, causal relationships cannot be established, and a study with sample randomization and a control group would be necessary to control for confounding variables that may interfere.

We initially considered the data collection to be satisfactory because many patients are reluctant to participate in research studies, and a high proportion of patients voluntarily took part in the first assessment. Nevertheless, substantial participant loss is common in survey- or questionnaire-based research, and in our study, we experienced a 32% attrition rate. These losses correspond mostly to patients who declined to complete the questionnaires a second time, mainly because the questionnaires were lengthy and caused considerable fatigue.

Additionally, data were obtained through patient self-report measures and clinician-based assessments, which may increase the risk of social desirability and interviewer bias. Patients who perceive themselves as being assessed may respond according to perceived social expectations.

The findings should be interpreted with caution due to the absence of correction for multiple testing. The sampling method was non-probabilistic and based on convenience, relying on the availability of patients referred to the program by their treating psychiatrists. As such, no comparison or control condition was available. Therefore, it cannot be conclusively demonstrated that the improvements observed were attributable solely to the rehabilitation program. Some degree of improvement could result from the natural progression following hospital discharge, particularly due to ongoing pharmacological treatment. As mentioned in the results section, all participants received psychopharmacological treatment and follow-up by their psychiatrist. The specific medications prescribed to each participant were not coded in this study. The main reason is that, during the prolonged stay in the rehabilitation program, adjustments in psychiatric medication were required for the patients.

An important limitation of this study is its retrospective pre-post design, the absence of a comparator group, and the substantial proportion of missing discharge data. Consequently, the findings should be interpreted with caution, as they do not allow for causal inference or attribution of the observed changes exclusively to the intervention program. The changes observed over time represent associations among participants with paired data and may reflect the combined influence of multiple concurrent factors, both related and unrelated to the intervention.

Despite these limitations, the study’s main strengths include the detection of clinically significant improvements, and the rigorous follow-up provided by mental health services.

### 4.2. Practical Implications and Future Research Directions

This study offers several practical implications. First, it underscores the importance of evaluating clinical processes and activities implemented in psychiatric rehabilitation services and disseminating research findings to the broader scientific community. Such efforts support an evidence-based practice framework, emphasizing critical and explicit use of the best available research evidence to guide clinical decision-making. Second, given the limited number of studies examining the benefits of psychiatric rehabilitation and testing specific programs, there is a clear need to continue implementing interventions aimed at improving health outcomes (e.g., self-care, social skills, psychoeducation). Finally, future research should include longitudinal designs to assess patients’ clinical progress after discharge and to determine whether improvements are maintained over time.

Moreover, subsequent studies could explore the differential impact of specific program components, such as cognitive rehabilitation, physical activity, or social skills training, to identify the most effective elements for diverse patient populations. Comparative studies involving control or alternative intervention groups would help clarify causal effects and enhance the generalizability of findings. Research integrating patient reported outcomes, quality of life measures, and cost effectiveness analyses could provide a more comprehensive understanding of the real-world value of psychiatric rehabilitation programs.

Furthermore, it would be highly relevant to explore the incorporation of new technologies, such as mobile applications, digital monitoring platforms, virtual reality, or telerehabilitation devices, to enhance adherence, and skill acquisition. Finally, multicenter studies across different regions and healthcare systems would be valuable to examine contextual factors influencing implementation, accessibility, and long term outcomes, ultimately guiding health policy and clinical practice.

## 5. Conclusions

The results demonstrate that a structured psychiatric rehabilitation program delivered within a partial hospitalization setting can generate meaningful improvements in key health outcomes among individuals with mental illness. Significant gains were observed in global functioning, heteroaggressive behavior, quality of life, and attitudes toward medication, reinforcing the central role of psychosocial and rehabilitative interventions in promoting recovery. These findings are consistent with previous research emphasizing the value of comprehensive, multidisciplinary rehabilitation models that integrate cognitive, behavioral, social, and occupational components to enhance adaptive functioning and support community reintegration [24,25]. Although reductions in suicide risk did not reach statistical significance, the observed clinical trend aligns with the heterogeneous results reported in the literature and highlights the need for specialized, targeted interventions when addressing suicidal behavior. Future research with larger samples and longitudinal follow-up is needed to further clarify the sustained impact of these interventions and the patient characteristics associated with optimal outcomes.

## Figures and Tables

**Table 1:** Programs and Group Sessions of the Partial Hospitalization Program.

Monday	Tuesday	Wednesday	Thursday	Friday
Activation Group	Activation Group	Activation Group	Activation Group	Activation Group
Creative Stimulation	Swimming Pool	Ergotherapy	Cognitive Rehabilitation and Supervised flats	Yoga And Body Awareness
Recreational Program				
Health Education	Community Integration	Interpersonal Communication/Social Skills	Cooking Workshop	Psychoeducation Group/ Illness Management
Lunch Program				

**Table 2:** Frequency and Percentage of Psychiatric Diagnosis according to ICD-10.

	Frequency	Percentage
F20	22	37.9
F25	6	10.3
F30	6	10.3
F40	1	1.7
F42	4	6.9
F44	1	1.7
F60	16	27.6
F90	1	1.7
UNDER STUDY	1	1.7
Total	58	100

**Table 3:** Results of the descriptive analysis of the quantitative variables of the study.

	*N*	Mean	*SD*	*N*	Mean	*SD*
		Pre Data			Pos Data	
Hetero-aggressive behavior	57	36.93	14.39	19	34.47	17.12
Suicide Risk	54	7.35	3.80	20	6.95	2.08
Global Assessment of Functioning (GAF)	56	42.68	15.19	46	69.13	25.10
Quality of Life	58	2.64	0.89	18	2.75	0.68
Attitude toward medication	58	8.14	1.24	19	8.39	1.20

*N*: Sample Size, *SD*: Standard Deviation.

**Table 4:** Results of Normality Tests.

	Shapiro–Wilk		
	Statistic	*df*	*p*
H-A B.-PRE	0.97	19	0.85
H-A B.-POST	0.95	19	0.55
SR-PRE	0.92	19	0.15
SR-POST	0.94	19	0.36
QL-PRE	0.97	18	0.90
QL-POST	0.94	18	0.39
DAI-PRE	0.95	19	0.43
DAI-POST	0.94	19	0.33
	**Kolmogorov–Smirnov**		
	**Statistic**	***df***	*** p ***
GAF-PRE	0.89	46	0.001
GAF-POST	0.91	46	0.003

H-A B.: Hetero-aggressive behavior; DAI: Attitude toward medication; SR: Suicidal Risk; QL: Quality of life, GAF: Global Assessment of Functioning, *df*: degrees of freedom, *p*: *p* value.

**Table 5:** Results of the inferential analysis of the quantitative variables of the study.

		*N*	Mean (*SD*)	Mean (*SD*)	Mean (*SD*) Paires Difference	*t*	*p*
			Pre Data	Post Data	Paired Samples Test		
Hetero-aggressive behavior		19	38.26(16.70)	34.47(17.12)	3.78 (7.75)	2.12	**0.047**
Suicide Risk		19	8(4.21)	7,1(4.08)	0.84(2.43)	1.50	0.07
Quality of Life		18	2.25(0.69)	2.75(0.68)	−0.50(0.86)	−2.48	**0.01**
Attitude toward medication		19	7.60 (1.31)	8.39(1.20)	−0.78 (1.15)	−2.97	**0.004**
		N	Average range	Sum of Ranks		Z	***p***
Global Assessment of Functioning (GAF)	NR	1	28.50	28.50		−4.80	**0.001**
	PR	35	18.21	637.50			
	Ties	10					

NR: negative ranks, PR: positive ranks, *N:* Sample Size, *SD*: Standard Deviation, *t: t* Student, *p: p* value.

**Table 6:** Matched Sample Effect Sizes.

			Confidence Interval 95%	
			Lower Limit	Upper Limit
Hetero-aggressive behavior	Cohen’s *d*	0.48	0.006	0.95
	Hedges Correction	0.46	0.006	0.91
Suicide Risk	Cohen’s *d*	0.35	−0.12	0.8
	Hedges Correction	0.33	−0.11	0.77
Quality of Life	Cohen’s *d*	−0.58	−1.07	−0.76
	Hedges Correction	−0.55	−1.03	−0.73
Attitude toward medication	Cohen’s *d*	−0.68	−1.17	−0.17
	Hedges Correction	−0.65	−1.12	−0.16

## Data Availability

The datasets generated and/or analyzed during the current study are not publicly available owing to the inclusion of confidential data but are available from the corresponding author upon reasonable request.

## Supplementary Materials

The following supporting information can be downloaded at: https://www.mdpi.com/article/10.3390/healthcare14020250/s1, File S1: Partial Hospitalization Programs.

## Abbreviations

The following abbreviations are used in this manuscript:

GAF	Global Assessment of Functioning
ICD	International Classification of Diseases
PHP	Partial Hospitalization Program
PR	Positive Ranks
*SD*	Standard deviation
NR	Negative Ranks
N	Sample Size
