# Supplementary material for: Impact of Psychiatric Rehabilitation on Chronicity and Health Outcomes in Mental Disorders: A Quasi-Experimental Study
Source: Healthcare (Basel). 2026 Jan 20;14(2):250. doi: 10.3390/healthcare14020250 (PMC12840731; doi:10.3390/healthcare14020250)
Supplement: Supplementary file 1 [file healthcare-14-00250-s001.zip › healthcare-4080008-supplementary.pdf]

Partial Hospitalization Programs

Partial hospitalization programs cover training and educational areas that form part of the comprehensive treatment for people with chronic mental illness.

These programs are:

1. Group Behavioral Activation Program

Behavioral activation is a psychological therapy that focuses on increasing individuals' engagement in activities that are pleasurable or provide a sense of achievement, in order to counteract inactivity and social isolation, which are highly prevalent in mental health conditions.

Occupational leisure profile questionnaires are used to explore interests, patterns, enjoyment, and barriers related to recreational activities. These questionnaires assess preferred activities (e.g., cinema, sports, reading), duration of engagement, balance with other daily tasks, level of enjoyment, health and financial factors, and the presence of friends or a supportive environment. Open-ended questions and standardized scales are employed to evaluate well-being, leisure time, motivation, and activity structuring.

This program is justified by the need to provide patients with a valuable and easy-to-implement therapeutic tool that promotes behavioral changes aimed at improving daily functioning and overall well-being.

In mental health patients, patterns of avoidance, social withdrawal, lack of motivation, apathy, inactivity, and loss of engagement in meaningful activities are commonly observed. Behavioral activation seeks to address these symptoms by helping patients reconnect with valued activities, restore healthy routines, and generate positive experiences that counteract negative emotional states.

The program is grounded in the premise that increasing engagement in rewarding and personally meaningful activities enhances the likelihood of experiencing positive emotions and improves motivation.

2. Activities of Daily Living (ADL) Program: This program refers to all aspects considered essential for adequate performance in behavioral areas related to self-care, personal competence, health, independence, and autonomy.

This area of work is justified because, in a high percentage of cases, the illness is accompanied by deterioration manifested in deficits in the areas addressed by the program.

The modules to be trained include, among others:

- Cooking workshop
- Shopping
- Household organization: care of personal belongings, organization and household tasks
- Nutrition
- Money management
- Use of transportation
- Completion of bureaucratic procedures
- Personal hygiene: self-care
- Health education: healthy habits (nutrition, sleep hygiene, first aid, sexuality)
- Orientation in the social environment (basic culture, knowledge of current events)

3. Cognitive Skills Rehabilitation Program:

The objective of this program is to train patients to recover, or at least prevent further deterioration of, their cognitive abilities (attention, memory, concentration, perception, spatial-temporal orientation, etc.) through rehabilitation, compensation, and repetition programs. Cognitive deficits have been well-documented in psychotic disorders.

These alterations, particularly attentional problems, are associated with significant losses in personal and social autonomy and increased vulnerability when facing stressful situations. These programs are based on the principle that a person with cognitive impairment has a certain degree of neuroplasticity and can improve if the necessary techniques and reinforcements are applied.

Intervention is carried out using **Gradior Suite**, a software program designed for the assessment, rehabilitation, and cognitive stimulation of individuals with mild cognitive impairment, acquired brain injury, neurological disorders, or normal aging. It allows for personalized interventions tailored to each user’s needs, with real-time monitoring and follow-up, while helping to maintain autonomy, slow cognitive decline, and promote patient well-being.

4. Social Skills Program:

This program constitutes a key aspect of psychosocial rehabilitation. One of the main problems faced by people with severe and long-term mental disorders (such as schizophrenia) is impaired social functioning, including difficulties in establishing interpersonal relationships, fulfilling social roles as appropriate, low participation in leisure activities, and limited enjoyment of activities. In fact, the

frequency of social contacts has been shown to be a good indicator of relapses and rehospitalizations. Similarly, one of the most important dimensions in measuring quality of life is the ability to participate in and enjoy social and leisure activities.

The objective of the program is to train individuals in acquiring and/or recovering behavioral repertoires (including facial expressions, eye contact, etc.), verbal skills (content of speech), paraverbal skills (affect, tone, volume of voice), and cognitive skills (thought processes, response time, amount of speaking) that provide the necessary tools for proper interpersonal functioning. This is considered a fundamental technique in psychosocial intervention and, as such, must be systematized and documented. The program aims to help individuals better face challenging situations, reducing stress levels and, consequently, the likelihood of relapses.

The program trains skills such as:

- **Verbal and non-verbal communication skills:** initiating a conversation, giving constructive criticism, greeting, etc.
- **Assertiveness**
- **Expression and recognition of emotions**
- **Behavioral rehearsal of specific situations or problems of each patient**

Methodology includes:

1. Identifying the need to learn a specific skill.
2. Structuring the skill into its different components and discussing them.
3. Modeling the activity through demonstration or role-playing.
4. Having the patient practice the activity through role-playing.
5. Providing feedback from the group and professionals on correctly performed steps.
6. Correcting areas that need improvement in future practice to ensure proper execution of the skill.
7. Repeating role-playing exercises until the skill is performed correctly.
8. Practicing the same with other patients.
9. Assigning homework to reinforce the skill.

This systematic approach ensures that patients gradually develop functional social skills applicable in real-life situations.

5. Psychoeducation and Illness Management Program:

This program is based on the idea that the patient should not be a passive recipient of treatment, but can and should actively participate in it. Its goal is to

equip patients with the knowledge and understanding of their condition so they can manage the phases of their illness, detect prodromal symptoms, prevent relapses, and cope with both symptoms and the stress caused by the illness.

These are group sessions where patients, by seeing themselves reflected in others and sharing symptoms and experiences, lose the sense of uniqueness and begin to question their own psychopathology. Accessible information is also provided, typically through psychoeducation courses, covering topics such as types of symptoms, medication and its benefits, side effects, and healthcare resources to turn to when needed.

A program of this kind is intended to improve adherence to medication and therapeutic guidelines. This is highly important both for preventing relapses and for reducing the need to use extensive healthcare network resources.

6. Socio-Community Integration Program:

This program is designed to promote socio-community participation by using community resources and developing social networks. Activities included in the program are:

- Current events program
- Basic knowledge group
- Bureaucratic procedures group
- Guidance and information on the use of mainstream educational and training resources

In many cases, this program is used as a motivating "hook" to engage users with the center. It is based on the principle of normalization and aims to encourage involvement in community social networks. There is also a specific program for collaboration and coordination with social services, in line with the objectives of the social-healthcare plan.

All activities at the Psychosocial Rehabilitation Center are guided by the principle of normalization, emphasizing the maximum possible use of community resources. In this context, the program provides knowledge of and access to community services. Finally, to the extent possible, participants are involved in awareness campaigns about mental illness and efforts to combat stigmatization.

7. Body Awareness Program:

This program works with patients through physical exercise, sports, yoga, and maintenance gymnastics, focusing on developing body awareness.

Body awareness is a fundamental therapeutic resource in addressing various mental health conditions because it allows the integration of physical, emotional, and cognitive experiences. Many psychological disorders manifest not only in thoughts and behaviors but also in bodily sensations, which are often unnoticed or misinterpreted. Promoting awareness of one's own body helps patients develop better emotional regulation, reduce internal tension, and strengthen their connection with immediate reality.

The body is the primary vehicle for emotional perception. Disorders such as anxiety, depression, trauma, and psychosomatic conditions are often accompanied by hypervigilance, body disconnection, or difficulty identifying physical sensations. Working on body awareness allows patients to recognize these early signals, facilitating preventive intervention and enhancing self-regulation.

Additionally, developing body awareness helps reduce physiological symptoms associated with stress, such as muscle tension, shallow breathing, or motor agitation. Techniques based on breathing, mindful movement, or relaxation can decrease sympathetic nervous system activation and promote states of calm and regulation.

Body awareness also strengthens the sense of identity and presence, which are often affected in conditions such as depression, dissociative disorders, or trauma-related effects. By learning to inhabit their own bodies, patients can improve self-esteem, internal control, and decision-making capacity from a more stable state.

Finally, this work promotes greater therapeutic adherence and active participation, as patients experience tangible changes and gain a better understanding of the relationship between their body, thoughts, and emotions.

8. Health School Program:

This program is aimed at patients with mental health disorders and is based on the need to promote educational processes that strengthen autonomy, self-care, and social integration for this population. Evidence shows that health education, when adapted to the cognitive, emotional, and social characteristics of users, significantly improves therapeutic adherence, reduces relapses, and enhances overall well-being.

People with mental health problems often face difficulties related to managing healthy habits, early recognition of symptoms, understanding their treatments, and actively participating in their own recovery process. Furthermore, this type of intervention helps reduce both internal and social stigma by promoting knowledge that supports inclusion and coexistence.

Overall, this Health School program for mental health patients is justified as an essential tool to improve quality of life, enhance functional recovery, and encourage active participation in the community, aligning with the principles of comprehensive, humanized, and person-centered care.

9. Occupational Therapy / Meaningful Occupation Program:

This program uses work, daily activities, manual tasks, and recreational activities to help people with mental health issues. It is an essential element for well-being, personal identity, and overall health. Its approach focuses not only on symptom management but also on improving functionality, promoting autonomy, and strengthening cognitive, emotional, and social skills that allow for a more independent and fulfilling life. This is particularly relevant in mental health, where difficulties are not always visible but can be deeply disabling.

Manual and recreational activities in occupational therapy are key tools for improving fine motor skills, cognition, and emotional expression through painting, drawing, origami, weaving, collage, jewelry making, and more. These activities benefit self-esteem, problem-solving skills, and provide strategies aimed at:

- Developing or recovering self-care, organization, household management, and time management skills.
- Retraining cognitive skills such as attention, planning, memory, and problem-solving.
- Promoting emotional stability, frustration tolerance, self-regulation, and self-esteem.
- Enhancing social skills and community participation, essential for preventing isolation and facilitating social integration.
- Reinforcing occupational identity, helping patients reclaim meaningful roles (worker, student, caregiver, active community member).
- Supporting preparation for work life, contributing to safe and sustainable socio-labor reintegration.

10. Creativity Program:

In the mental health field, creative activities—such as artistic expression, writing, music, or movement—promote emotional regulation, communication, and psychological well-being. Creativity is a fundamental human capacity that enables the generation of new ideas, the expression of emotions, and the construction of personal meaning.

Implementing a creative stimulation program represents a complementary therapeutic intervention of high clinical value. Patients with mental health

disorders often experience difficulties in areas such as motivation, emotional expression, social interaction, problem-solving, self-esteem, and stress management. Creative stimulation provides a safe, non-threatening space to work on these aspects, facilitating symbolic expression of internal experiences and promoting greater self-understanding. It also encourages active patient participation, reinforces self-efficacy, and supports treatment adherence.

From a neuropsychological perspective, creative activities activate brain networks associated with cognitive flexibility, sustained attention, working memory, and sensory integration. This can help improve cognitive functions often impaired in various mental disorders, such as depression, schizophrenia, and anxiety disorders. Emotionally, artistic creation has been shown to reduce cortisol levels and enhance experiences of flow, relaxation, and well-being.

In institutional or community contexts, these programs promote group cohesion and socialization, reducing feelings of isolation and stigmatization. Group creative work helps develop social skills, practice cooperation, and foster a sense of belonging—all essential elements for psychosocial recovery.

Finally, creative stimulation can be easily adapted to each patient's needs, abilities, and personal pace, making it an inclusive, flexible, and respectful tool for the diversity of clinical experiences.

Justification for the Use of a Heteroaggressiveness Scale in a Partial Hospitalization Program

Heteroaggressiveness is a psychological concept that refers to an individual's tendency to express aggression toward other individuals or groups. It is characterized by hostility, verbal or physical violence, and a lack of empathy toward others.

In this context, it is important to use a standardized scale for the assessment of heteroaggressiveness that allows for the identification of the presence, intensity, and frequency of such behaviors. The use of this specific instrument enables the timely detection of risks, making it possible to implement preventive and therapeutic interventions.

The objective assessment of aggressiveness in psychiatric patients is very useful for distinguishing chronically aggressive patients from those who present aggressive episodes following the onset or exacerbation of psychotic symptomatology.

The heteroaggressiveness scale helps in decision-making within the therapeutic rehabilitation plan by providing criteria for behavioral management, treatment

planning, resource allocation, and the evaluation of the patient's progress throughout the rehabilitation process.

The objective assessment of aggressiveness in psychiatric patients is very useful for distinguishing chronically aggressive patients from those who present aggressive episodes following the onset or exacerbation of psychotic symptomatology.

Therefore, the implementation of a heteroaggressiveness scale in a mental health hospital is a tool to optimize the quality of care and to promote rehabilitation processes.

Justification for the Use of an Attitude Toward Medication Scale in a Partial Hospitalization Program

The justification for using an attitude toward medication scale in a partial hospitalization (PH) program lies in its ability to assess the patient's beliefs, perceptions, and willingness regarding their pharmacological treatment. This assessment is important for predicting therapeutic adherence, identifying barriers, personalizing the care plan, improving emotional well-being, and, above all, optimizing mental health treatment outcomes.

Treatment adherence is vital in the psychiatric rehabilitation process, as it is directly related to clinical stability, reduction of relapses, psychosocial functioning, and patients' quality of life.

In psychiatric rehabilitation hospitals, where treatments are often long-term, systematically evaluating adherence is essential to optimize therapeutic outcomes.

The implementation of a treatment adherence scale provides an objective and standardized tool to identify the patient's level of compliance with pharmacotherapy, attendance at individual mental health appointments, and participation in programs. This measurement allows for the early detection of low-adherence behaviors, which are associated with a higher risk of relapse, readmission, and functional deterioration.

Likewise, the use of a validated scale supports clinical decision-making by enabling the interdisciplinary team to design individualized intervention strategies, work on illness awareness, improve the therapeutic alliance, and enhance patient autonomy.

In the context of psychiatric rehabilitation, lack of insight, residual symptoms, stigma, and cognitive difficulties can affect therapeutic compliance. Therefore,

the use of an adherence scale is justified as an essential resource for comprehensive patient assessment. The scale acts as an initial diagnostic tool to understand the patient's starting point.

Justification for the Use of a Quality of Life Assessment Scale in a Partial Hospitalization Program

The assessment of quality of life constitutes an essential component in mental health hospitals, as the goal of treatment is not only the reduction of psychopathological symptoms, but also the promotion of overall well-being, functional autonomy, and social reintegration. In this context, the implementation of a quality of life assessment scale is fundamental in order to obtain a comprehensive view of the outcomes of therapeutic interventions.

The quality of life assessment scale allows the measurement of key dimensions such as physical, psychological, and emotional well-being; interpersonal relationships; level of independence; social participation; and the subjective perception of life satisfaction. These items are especially relevant in patients with severe mental disorders, who often experience significant limitations in daily functioning and community integration.

The use of a standardized scale facilitates the identification of individual needs, strengths, and areas for improvement, thereby contributing to the planning of personalized, person-centered interventions. It also allows for monitoring the patient's progress throughout the rehabilitation process, evaluating the effectiveness of therapeutic programs, and making adjustments to individualized treatment plans.

Assessing quality of life provides meaningful information for clinical decision-making and promotes active patient participation by recognizing the patient's perception and experience as central elements of the therapeutic process.

In conclusion, the quality of life assessment scale in a mental health hospital is an indispensable tool for ensuring comprehensive care oriented toward functional and social recovery, contributing to the improvement of overall well-being and the quality of life of treated individuals.

Justification for the Use of the Plutchik Suicide Risk Scale in a Partial Hospitalization Program

The Plutchik Suicide Risk Scale is a validated and widely used instrument for the early detection of suicide risk, which is especially relevant in the context of a mental health hospital, where patients present chronic or severe mental disorders, a history of self-harming behaviors, and complex psychosocial factors.

The implementation of this scale allows for a structured assessment of key aspects associated with suicide risk, such as previous attempts, suicidal ideation, hopelessness, impulsivity, depression, and lack of support networks, providing an objective score that facilitates the identification of the level of risk. This information is essential for planning therapeutic interventions tailored to the patients' clinical needs.

In the field of mental health, where the therapeutic process is continuous and long-term, the Plutchik Scale is particularly useful due to its simple, quick, and easy-to-understand application, allowing for periodic use to monitor changes in the patient's emotional state throughout treatment. This enables the early detection of decompensation.

In conclusion, the Plutchik Suicide Risk Scale is an effective tool in the mental health hospital setting, as it facilitates the timely detection of suicide risk, guides clinical decision-making, and contributes significantly to suicide prevention.

Justification for the Use of the Global Assessment of Functioning Scale in a Partial Hospitalization Program

The Global Assessment of Functioning (GAF) Scale is a clinical instrument used to comprehensively assess the level of psychological, social, and occupational functioning of individuals with mental disorders. In the context of a mental health hospital, its application is particularly relevant, as patients often present significant impairments in overall functioning as a result of chronic or severe mental disorders.

The use of this scale allows for a quantitative and standardized assessment of the patient's level of functioning, taking into account symptom severity, functional loss, and the impact on activities of daily living. This information is essential for establishing a baseline, identifying areas of greater impairment, and setting personalized therapeutic goals within the rehabilitation process.

Within the framework of psychiatric rehabilitation, whose objectives include promoting functional recovery and social reintegration, the Global Assessment of Functioning Scale allows for monitoring the patient's improvement over time.

In conclusion, the Global Assessment of Functioning Scale is a useful and relevant tool in the mental health hospital setting, as it enables the assessment of the patient's overall functioning, guides the therapeutic process, and measures progress achieved during rehabilitation.
